# Knowledge translation following the implementation of a state-wide Paediatric Sepsis Pathway in the emergency department- a multi-centre survey study

**DOI:** 10.1186/s12913-021-07128-2

**Published:** 2021-10-26

**Authors:** Amanda Harley, Luregn J. Schlapbach, Paula Lister, Debbie Massey, Patricia Gilholm, Amy N. B. Johnston

**Affiliations:** 1 School of Nursing, Midwifery and Social Work, The University of Queensland, Brisbane, QLD Australia; 2 Child Health Research Centre, The University of Queensland, and Paediatric Intensive Care Unit, Queensland Children’s Hospital, QLD Brisbane, Australia; 3 Department of Emergency Medicine, Gold Coast University Hospital, Gold Coast, QLD Australia; 4 Pediatric and Neonatal Intensive Care Unit, Children’s Research Center, University Children’s Hospital Zurich, Zurich, Switzerland; 5 Paediatric Intensive Care Unit, Sunshine Coast University Hospital, Sunshine Coast, QLD Australia; 6 School of Medicine, Griffith University, Brisbane, QLD Australia; 7 School of Nursing and Midwifery, Southern Cross University, Gold Coast, QLD Australia; 8 Department of Emergency Medicine, Princess Alexandra Hospital, Brisbane, QLD Australia

**Keywords:** Clinical pathway, Implementation, Knowledge translation, Management, Nurse, Recognition; sepsis; sepsis pathway; septic shock

## Abstract

**Background:**

Several health care systems internationally have implemented protocolised sepsis recognition and treatment bundles for children to improve outcomes, as recommended by the Surviving Sepsis Campaign. Successful implementation of clinical pathways is challenging and dependent on nurse engagement. There is limited data on knowledge translation during implementation of sepsis quality improvement programs.

**Methods:**

This cross-sectional, multicentre observational survey study evaluated knowledge and perceptions of Emergency Department nurses in relation to the recognition, escalation and management of paediatric sepsis following implementation of a sepsis pathway. The study was conducted between September 2019 and March 2020 across 14 Emergency Departments in Queensland, Australia. The primary outcome was a sepsis knowledge score. An exploratory factor analysis was conducted to identify factors impacting nurses’ perceptions of recognition, escalation and management of paediatric sepsis and their association with knowledge. Using a logistic mixed effects model we explored associations between knowledge, identified factors and other clinical, demographic and hospital site variables.

**Results:**

In total, 676 nurses responded to the survey and 534 were included in the analysis. The median knowledge score was 57.1% (*IQR* = 46.7–66.7), with considerable variation observed between sites. The exploratory factor analysis identified five factors contributing to paediatric sepsis recognition, escalation and management, categorised as 1) knowledge and beliefs, 2) social influences, 3) beliefs about capability and skills delivering treatment, 4) beliefs about capability and behaviour and 5) environmental context. Nurses reported strong agreement with statements measuring four of the five factors, responding lowest to the factor pertaining to capability and skills delivering treatment for paediatric sepsis. The factors knowledge and beliefs, capability and skills, and environmental context were positively associated with a higher knowledge score. Years of paediatric experience and dedicated nurse funding for the sepsis quality improvement initiative were also associated with a higher knowledge score.

**Conclusion:**

Translation of evidence to practice such as successful implementation of a sepsis care bundle, relies on effective education of staff and sustained uptake of protocols in daily practice. Our survey findings identify key elements associated with enhanced knowledge including dedicated funding for hospitals to target paediatric sepsis quality improvement projects.

**Supplementary Information:**

The online version contains supplementary material available at 10.1186/s12913-021-07128-2.

## Background

Sepsis, defined as a dysregulated host response to infection, remains one of the leading causes of preventable death and disability for children globally, with recent incidence estimates exceeding 25 million for the paediatric age group [1, 2]. In high-income countries, approximately one third of sepsis deaths occur in previously healthy children [3], and about one third of survivors will manifest ongoing sequelae [4] which impact families, healthcare facilities, and the community. In response to the global burden of paediatric sepsis the World Health Organisation (WHO) declared sepsis a global priority [5], prompting Australian professional bodies to develop a National Sepsis Action Plan to improve awareness, recognition and management of sepsis [6–8].

The paediatric Surviving Sepsis Campaign (SSC) recommends the use of a sepsis protocol and systematic screening tool to assist clinicians in the early recognition and management of sepsis in children [9]. Observational data have demonstrated benefit in a protocolised approach to sepsis in children, however significant barriers exist preventing the application of pathways in clinical practice that results in substantial variability in care both nationally and internationally [6, 10–14]. For example, the United Kingdom (UK) reported varied median implementation rates between 22 and 47% for components of the sepsis care bundle across 160 hospitals involved in sepsis pathway implementation [15]. The appointment of a sepsis board and a Patient Safety Facilitator resulted in improved pathway compliance in a four-year pilot program in London [16]. The largest paediatric sepsis Quality Improvement (QI) initiative in New York State revealed variability in the delivery of the mandated sepsis bundle [10]. Yet, to date there are limited data on the factors associated with success in implementation of sepsis QI programs, the processes related to successful knowledge translation (KT) and subsequent changes in behaviour [17, 18]. KT is a multidimensional process, whereby knowledge translates to evidence application, aiming to improve patient outcomes [19]. Recent studies have combined self-reported perceptions and participant knowledge as measures of KT [20]. Applying a KT methodology presents an opportunity to review, refine and improve implementation of clinical guidelines and reduce variability in care by knowledge generation, which when applied to clinical practice, may enhance uptake of evidence based practice [21, 22]. Implementation of a sepsis clinical pathway is an example of a KT intervention, which can be enhanced by evaluation [23]. Effective KT should include evaluation that assesses and explores underpinning factors to guideline use [21], and KT frameworks, such as the Theoretical Domains Framework (TDF), have been designed for this purpose [24]. The TDF identifies key factors supporting behaviour change in clinicians to inform strategies to enhance the uptake of evidence-based practice [21, 22, 25]. Once factors associated with the utilisation of evidence-based pathways are identified, interventions can be designed to increase KT [26].

Research conducted in the ED aims to advance knowledge, translating to improved practice and healthcare delivery. Despite this aim, such evidence is not always utilised [27]. Involving key knowledge users (nurses) is imperative to ensure pathways are integrated into practice [27]. Little is known about KT and sepsis pathways [28] and no known KT studies exist focusing on paediatric sepsis and nurses. Nurses’ perceptions and engagement are key to successful KT [26] of clinical pathways. To advance knowledge and application of evidence-based practice, we introduced a Paediatric Sepsis Pathway (PSP), including targeted education for nurses on paediatric sepsis recognition, escalation and management, and on how to translate the PSP into practice. Nurses are the first point of patient contact in the ED, responsible for both initial and ongoing assessment [29, 30]. Nurses are optimally positioned to recognise, escalate and manage sepsis, and are essential to pathway implementation [31, 32]. The aim of this study was to evaluate the implementation of the PSP in participating Emergency Departments (ED) across the state of Queensland, Australia. This was achieved through a multi-centre survey of ED nurses’ knowledge and perceptions that explored key elements of the PSP, reflecting KT. Specifically, we aimed to identify factors impacting nurses’ perceptions of paediatric sepsis recognition, escalation and management and their association with knowledge, underpinned by the TDF.

## Methods

### Overview

We performed a multi-centre, cross-sectional survey assessing knowledge and perceptions of nurses working in 14 EDs following the implementation of a PSP in Queensland, Australia. Ethical approval was provided by Children’s Health Queensland Human Research Ethical Committee (HREC/18/QRCH/167) and ratification from The University of Queensland (20190000093). This study followed the Consensus-Based Checklist for Reporting of Survey Studies (CROSS) [33].

Research in healthcare typically aims to progress knowledge, translating to improved health outcomes [27]. For this reason, the study aim was to i) determine nursing knowledge of paediatric sepsis recognition, escalation and management following implementation of a PSP, and to ii) explore the factors contributing to that knowledge, established from nurses’ perceptions reflecting the key elements of the PSP. Nurse’ knowledge score was generated from responses to questions formulated from content and implementation components of the PSP and was used as a proxy marker for KT, in combination with the factors reflecting clinical engagement with the PSP. Convenience sampling was used to recruit eligible participants and the large cohort, including widespread dissemination within each site and across the state of Queensland, aimed to result in a representative sample. Eligible participants were nurses working in participating EDs where the PSP had been implemented for a minimum period of 3 months and the nurses must have cared for paediatric patients in their current ED for a minimum of six months.

Fourteen hospitals across Queensland participated in a state-wide QI project that implemented a PSP in the ED from August 2018 to December 2019 and were included in the present study. The majority of Queensland EDs treat adult and paediatric populations (mixed ED) and nurses are trained to care across the lifespan [34]. Sites included four large, dedicated paediatric EDs and 10 mixed EDs, including metropolitan and regional facilities, with a combined nursing workforce of *n* = 1796. The funding allocated per site for the sepsis QI project was described as ‘sepsis nurse funding’; this is the product of the designated nurse salary (weighting) and the full time equivalent in months spent in the role [35, 36]. ‘Sepsis nurse funding’ varied in amounts and duration (Supplementary Material 1).

### Survey design

The survey was designed building on the Queensland state-wide PSP (Supplementary Material 2) as a framework to align with the key elements of recognition, escalation and management of paediatric sepsis [9].

The survey had three sections comprising 48 items (Supplementary Material 3). The first section contained demographic questions. The second section surveyed nurse’s knowledge of paediatric sepsis acquired from the PSP, including 15 multiple choice questions (with pre-defined single or multiple correct answer options). The third section investigated factors impacting nurses’ perceptions of paediatric sepsis recognition, escalation and management, contributing to evaluation of the implementation of a PSP. This section consisted of 25 statements where nurses responded to whether they agreed with the statements on a seven-point Likert scale ranging from one (strongly disagree) to seven (strongly agree) and were developed from the TDF. The TDF is a validated theoretical framework, developed from multiple behaviour change theories and provides insight into evidence for factors that influence clinical practice. The survey design was specifically informed by the Determinants of Implementation Behaviour Questionnaire (DIBQ), which is a validated TDF questionnaire targeting clinician implementation behaviour [25], providing insight into PSP utilisation and KT to practice.

### Content, face, construct validity and reliability

Expert review, coupled with findings from reviewed literature, ensured the survey questions were formulated to achieve maximal authenticity (AH, LJS). Correct (knowledge) responses were formulated from the current Queensland PSP. The survey was piloted by 30 multidisciplinary content experts including medical specialists, ED nurses and medical officers, academics, those in leadership roles within the state-wide project and end users to ensure face, content and construct validity [25, 37]. Time to complete the survey was, on average, 13 min [37]. Pilot feedback identified expected responses and a high level of user acceptability, indicating a high degree of content validity. Based on the pilot, minor modifications were made to ensure questions were clear and appropriate to context, consensus reflected evidence-based practice.

The survey underwent reliability testing and was designed to permit an exploratory factor analysis (EFA) to explore the participants’ perceptions section of the survey; to determine the factors that were present in relation to the recognition, escalation, and management of sepsis and their association with the survey primary outcome (knowledge score).

### Survey dissemination and data collection

In each site a designated nurse was responsible for implementing the pathway including leading the QI initiative locally and participating in the broader state-wide program, provision of local education and collecting data. Survey commencement was guided by local governance approvals and eligibility criteria (Supplementary Material 1). The surveys were distributed over an approximately six-week period via an electronic platform, QR code linkage and on paper, which was led locally with support by the central research team. A standardised introduction to the survey was distributed to participants at each site prior to survey commencement to ensure consistency. Surveys were completed anonymously, containing no individual identifiers to maintain confidentiality. Where participants voluntarily provided an email address and were observed to not have completed the survey (as per RedCap) they were sent one reminder email promoting completion by the central team who were unaware of the participant or their results. Data were transcribed from paper where required and stored on a secure RedCap database. Sites were de-identified for confidentiality and are referred to as sites 1–14.

### Exploratory factor analysis

Previous researchers have utilised theoretical frameworks to elucidate and assess factors that influence behaviour change [38] whereby design, application and interpretation is tailored to specific environments [39]. The EFA identified five factors which were categorised and adapted to fit the specific ED context, whereby allocation and alignment reflects the key constructs of the TDF [16, 39]. The five identified factors had good internal consistency measured by Cronbach’s alpha, α > 0.7. The factor loadings for each item, corresponding questions and labelled factors, are displayed in Table 1. An individual’s score on each factor was operationalised using factor scores. The factor scores were used in the main regression analyses to investigate their association with the primary outcome. Additional detail on the EFA and factor score methods is contained in Supplementary Material 4.

**Table 1:** Median response (IQR) and factor loadings for the five factors identified in the exploratory factor analysis for the broad nursing cohort. Only loadings greater than 0.3^a^ are displayed. Cronbach’s alpha (α) is reported for each factor

Question^a^	Median response (IQR)	Factor 1: Knowledge and beliefs about paediatric sepsis and pathway application (α = 0.89)	Factor 2: Social influences when recognising, escalating and managing paediatric sepsis (α = 0.83)	Factor 3: Beliefs about capability and skills delivering treatment for paediatric sepsis (α = 0.84)	Factor 4: Beliefs about capability and behaviour in recognising, escalating and managing paediatric sepsis (α = 0.87)	Factor 5: Environmental context and resources in the ED for recognising, escalating and managing paediatric sepsis (α = 0.74)
24. I am confident that I could recognise sepsis in a paediatric patient	6 (5–6)	–	–	–	0.70	–
26. I am sufficiently trained to respond to sepsis in a paediatric patient	6 (5–6)	–	–	–	0.63	–
27. I have a clear plan of how I will escalate care of a patient with suspected sepsis following the PSP	6 (6–7)	–	–	–	0.60	–
28. I am confident that I can set up an Adrenaline infusion in a time critical situation	5 (3–6)	–	–	0.75	–	–
29. I have the skills to calculate and deliver a fluid bolus to a paediatric patient as rapidly as their condition demands	6 (5–6)	–	–	0.92	–	–
30. When I have to prescribe or deliver an intravenous antibiotic I feel comfortable using the guide on the PSP	6 (5–7)	–	–	0.50	–	–
31. Escalating my concerns to a Senior Medical Officer that a child could have sepsis is	6 (6–7)	–	0.66	–	–	–
33. For me, seeking support from colleagues when uncertain about the management of paediatric sepsis is	6 (6–7)	–	0.70	–	–	–
35. My hospital provides doctors and nurses with sufficient training to recognise and manage treatment for paediatric patients with sepsis	6 (5–6)	–	–	–	–	0.48
36. Senior staff in the organisation in which I work are willing to listen to my problems with escalating care and finding placement for paediatric patients with sepsis	6 (6–6)	–	0.70	–	–	–
37. I can count on gaining support from doctors and nurses to respond when I suspect a patient has sepsis	6 (6–7)	–	0.81	–	–	–
38. I can remember the important steps of care for paediatric patients with sepsis	6 (5–6)	–	–	–	0.49	–
39. In my ED, I think there are all the necessary resources available to efficiently manage paediatric sepsis	6 (5–6)	–	–	–	–	0.55
40. In my hospital, there is a good collaboration between Paediatrics or retrieval teams and the ED when accepting patients with sepsis who require ongoing care	6 (5–6)	–	–	–	–	0.49
41. Delivering sepsis care following the pathway is a high priority of mine when working in the ED	6 (6–7)	0.53	–	–	–	–
43. I am often triggered to think about sepsis in children by the level of parental concern voiced	6 (5–6)	0.58	–	–	–	–
44. My ED recommends the inclusion and involvement of parents in assessment and clinical management of paediatric patients with sepsis	6 (6–7)	0.54	–	–	–	–
47. If I deliver sepsis care following the PSP I will feel satisfied in the care I am delivering to my patient	6 (6–7)	0.77	–	–	–	–
48. Delivering the treatment bundle on the sepsis pathway is part of my role as a clinician	6 (6–7)	0.77	–	–	–	–
49. If I deliver care within one hour following the treatment bundle on the sepsis pathway this will result in a better patient outcome.	7 (6–7)	0.80	–	–	–	–
50. Delivering sepsis care following the PSP is something I do as part of routine patient management	6 (6–7)	0.68	–	–	–	–

^a^ To ease interpretation, only factor loadings greater than or equal to 0.3 are displayed, corresponding to the cut-off used to determine whether an item contributed to a factor, see Supplementary Material 4 for details

### Statistical analyses

The primary outcome, the knowledge score, was calculated for each participant as the proportion of correct responses on the knowledge section of the survey (questions 8 to 23). More detail on scoring and processing of survey data, such as accounting for missing data, is contained in Supplementary Material 5 and 6.

All demographic details were summarised using descriptive statistics, including counts and proportions. A binomial logistic mixed effects model was used to explore the associations between the factor scores, demographics, and the hospital site variables on the knowledge scores [40]. A random intercept of site was incorporated into the model to determine if the variation in knowledge scores could be attributable to hospital (site).

The demographic variables, comprising questions 3 to 7 of the survey, were initially explored to be included as additional covariates in the model. However, paediatric experience, ED experience and nursing experience were found to be highly correlated (*r* = 0.71 to 0.81), so to avoid issues of multicollinearity, only paediatric experience was included in the model due to the population of interest, paediatrics [41]. Therefore, the demographic variables included in the model were age, years of paediatric experience and self-reported frequency of exposure to caring for a child with sepsis. Site variables included were ‘sepsis nurse funding’, (Supplementary Material 1), and the number of clinicians working within each ED. Models including and excluding the random intercept were compared and including the random intercept significantly improved the model fit. The model reported contains the demographic and site variables, the random intercept for hospital site and the five factor scores. All statistical analyses were undertaken using R (version 4.0.2) [42].

## Results

### Primary outcome – respondent’s knowledge

In total, 1796 nurses were invited to complete the survey, 676 nurses responded, 544 answered 80% or more of the survey and 534 completed the demographic questions and were included in the final analysis. The combined response rate for all respondents was 37.6%, individual site response rates are reported in Supplementary Material 1. The majority of survey respondents reported working in nursing for 10+ years (39%, *n* = 207), and were aged between 26 and 30 years (28%, *n* = 151). 31% (*n* = 166) of respondents reported exposure to paediatric sepsis on a monthly basis. Respondent characteristics are described in Table 2.

**Table 2:** Respondent characteristics

Characteristic	***N*** = 534^a^ (% of total sample)
Hospital	
1	27 (5.1%)
2	26 (4.9%)
3	66 (12%)
4	94 (18%)
5	19 (3.6%)
6	70 (13%)
7	16 (3.0%)
8	51 (9.6%)
9	6 (1.1%)
10	33 (6.2%)
11	41 (7.7%)
12	35 (6.6%)
13	33 (6.2%)
14	17 (3.2%)
Nursing role ^b^	
Clinical facilitator	13 (2.4%)
Enrolled nurse	4 (0.7%)
Endorsed enrolled nurse	13 (2.4%)
Registered nurse	379 (71%)
Clinical nurse	105 (20%)
Nurse unit manager	1 (0.2%)
Nurse educator	7 (1.3%)
Clinical nurse consultant	10 (1.9%)
Nurse practitioner	8 (1.5%)
Research nurse	5 (0.9%)
Other	3 (0.6%)
Age	
20–25 years	72 (13%)
26–30 years	151 (28%)
31–35 years	94 (18%)
36–40 years	66 (12%)
41–50 years	93 (17%)
51–60 years	49 (9.2%)
61 + years	9 (1.7%)
Years of nursing experience	
6–11 months	19 (3.6%)
1–3 years	84 (16%)
4–6 years	138 (26%)
7–9 years	84 (16%)
10+ years	207 (39%)
Unknown	2
Years of paediatric experience	
6–11 months	69 (13%)
1–3 years	149 (28%)
4–6 years	125 (23%)
7–9 years	76 (14%)
10+ years	115 (22%)
Years of ED experience	
6–11 months	45 (8.5%)
1–3 years	149 (28%)
4–6 years	130 (25%)
7–9 years	81 (15%)
10+ years	124 (23%)
Unknown	5
Self-reported frequency of exposure to paediatric sepsis	
Weekly	88 (16%)
Fortnightly	101 (19%)
Monthly	166 (31%)
Six monthly	71 (13%)
Yearly	13 (2.4%)
Don’t know	95 (18%)

^a^Statistics presented: n (%)

^b^ All nurses listed are registered with the Nursing and Midwifery Board to practise and categories listed are for Queensland, Australia. Enrolled and Endorsed eErolled nurses have completed a certificate IV or a Diploma in Nursing and work under supervision of a Registered Nurse (RN). The subsequent categories listed are all RNs, known as Nursing Grade 5 (NG5) and have completed a three year bachelor degree in nursing. Clinical Nurses, Clinical Facilitators and Research Nurses (NG6) are one grade level above an RN. Clinical Nurse Consultants, Nurse Unit Managers and Nurse Educators (NG7) are two grade levels above an RN. NG6 and NG7s have been working in their specialised field for a number of years and are advised to be enrolled or have completed post-graduate studies. Nurse Practitioners are independent clinicians that have completed a masters of Nurse Practitioner [1]

**Fig. 1:**
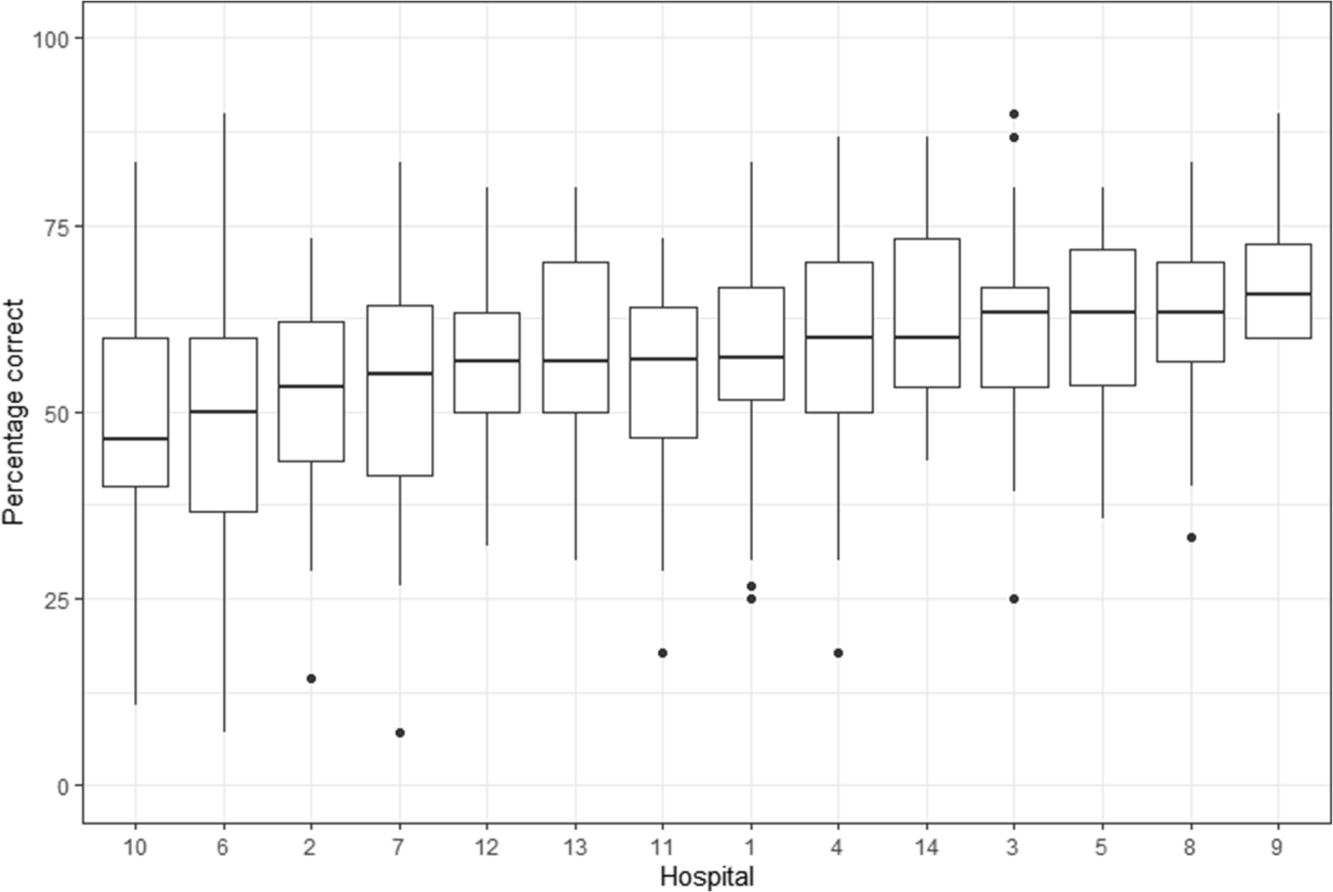
Distribution of knowledge scores per hospital. Sites have been arranged in order of median response per hospital. The centre line for each box plot is the median value, the upper-lower limits of the box are the 1st and 3rd quartile. The whiskers correspond to the maximum and minimum points that are 1.5* the IQR from the 1st and 3rd quartiles. Data that exceed these limits are considered outliers and plotted individually

The median percentage of correct responses for the knowledge section of the survey was 57.1% (*IQR* = 46.7–66.7). Considerable site variability for knowledge scores was observed, with the median percentage of correct responses per site ranging from 46.4 to 65.8%, **see** Fig. 1. Overall, the median proportion of correct responses for sepsis recognition was 50.0% (*IQR* = 37.5–62.5), for escalation 50.0% (*IQR* = 25.0–75.0), compared to 62.5% (*IQR* = 50.0–72.2) for management (Supplementary Material 7).

### Self-reported pathway utilisation

The questions exploring implementation of the PSP (Supplementary Material 8) indicated that 96% (*n* = 513) of nurses utilised the PSP to guide sepsis management. The PSP was cited as the main resource used to guide treatment including antibiotic administration (89%, *n* = 475) and preparation of an adrenaline infusion (68%, *n* = 363).

**Fig. 2:**
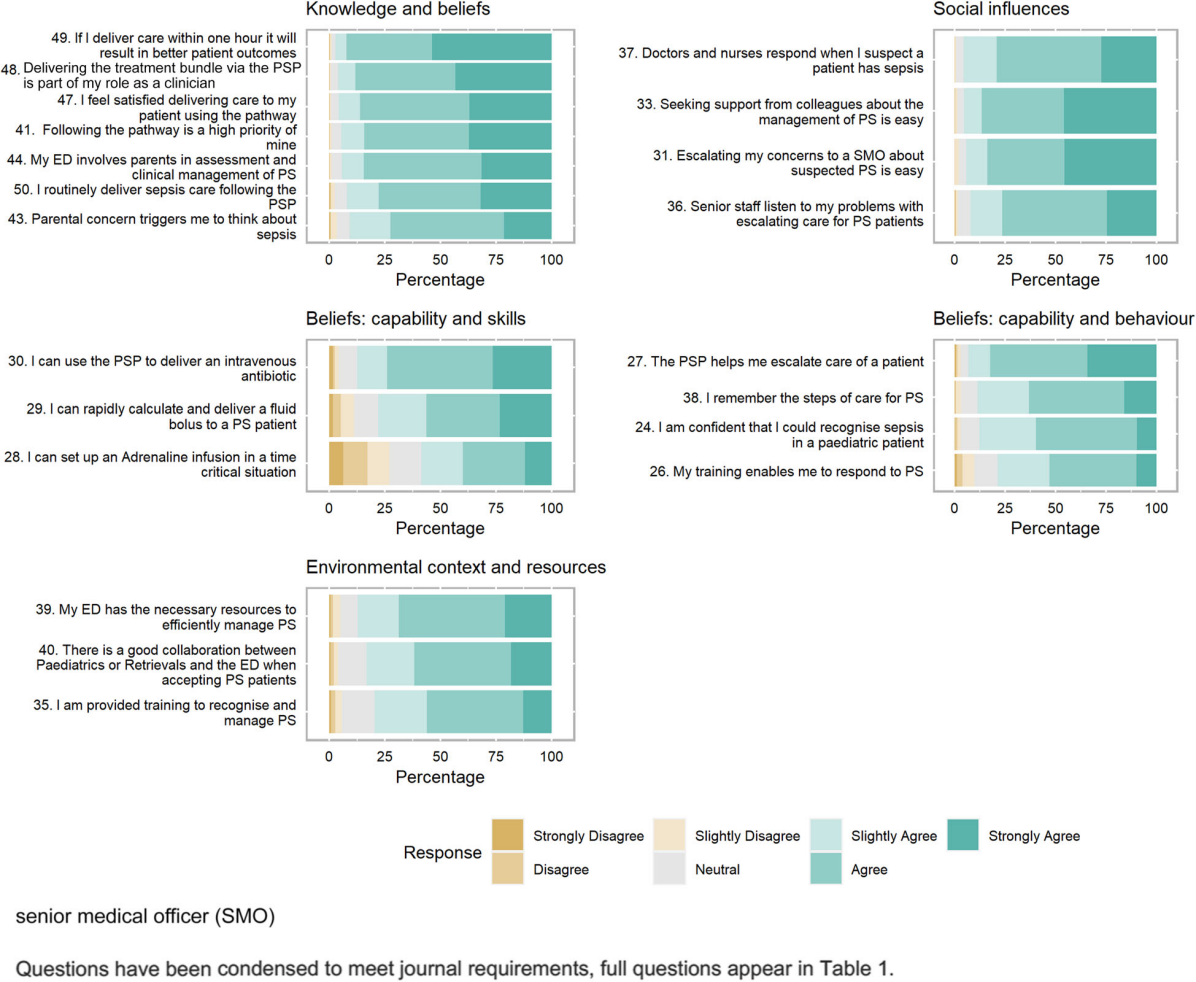
Response percentage for questions that comprise each factor. Each subfigure comprises the statements that belong to each of the five factors. The bars represent response percentages with green colours indicating positive agreements responses, brown colours indicating disagreement responses and grey indicates neutral responses

The seven-point Likert scale scores for each of the statements comprising the five factors associated with the pathway utilisation are displayed in Fig. 2. The median Likert scale response for statements related to knowledge and beliefs (Factor one), social influences (Factor two), beliefs about capability and behaviour (Factor four) and environmental context and resources (Factor five) were very high on the scale; ranging from 6 to 7 (*IQR =* 5–7). Factor three, beliefs about capability and skills, scored lower with a median response of 5–6 (*IQR* = 3–7). The median response for each individual question is displayed in Table 1. Site variation for each of the five factors is displayed in Supplementary Material 9.

### Variables associated with knowledge about sepsis

**Fig. 3:**
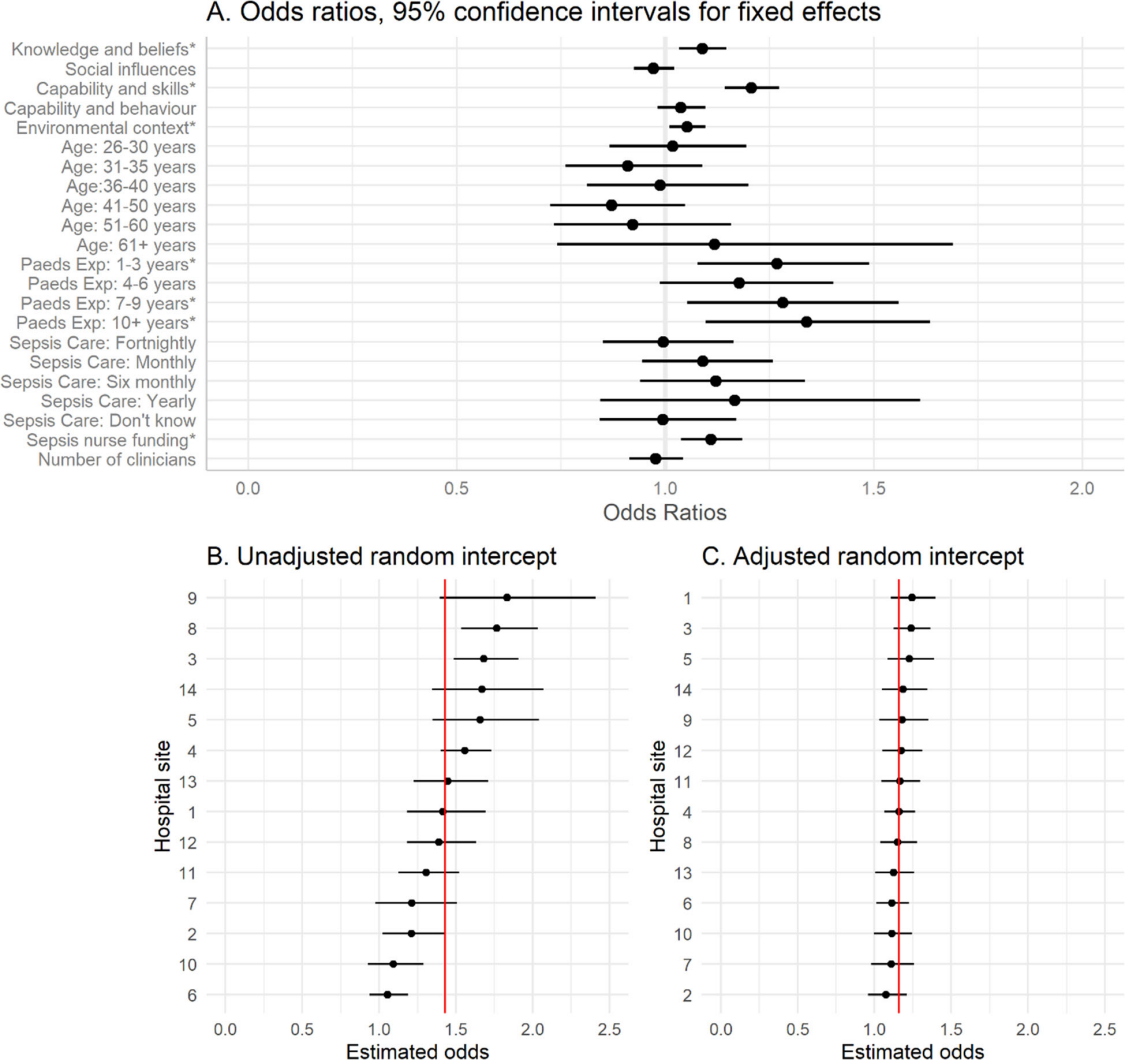
**A** Odds ratios and 95% Confidence intervals for the impacts of the fixed effects of the model on knowledge scores. Variables marked with * are statistically significant (*p* < 0.05; see Table 2). The reference category for age is 20–25 years. The reference category for paediatric experience is 6–11 months and the reference category for sepsis care is weekly. **B** Estimated unadjusted random intercept and 95% CI per hospital site. Hospitals (1–14) have been sorted by size of estimated odds of correct response. The red solid vertical line indicates the population estimated intercept. **C** Adjusted random intercept and 95% CI per hospital site. Hospitals (1–14) have been sorted by size of estimated odds of correct response. The red solid vertical line indicates the population estimated intercept. Panel **C** shows the variation between hospitals after adjusting for fixed effects including factors, sepsis nurse funding and years of paediatric experience

The results of the binomial mixed effects model used to investigate the effects of the five factors, demographic, clinical and site level variables on knowledge score, including a random intercept for hospital site to account for variation in knowledge scores between sites are displayed in Table 3. Three of the five factors had a significant and positive effect on knowledge scores, these included (a) knowledge and beliefs (*OR* = 1.09; *p* = 0.001), (b) beliefs about capability and skills (*OR* = 1.21; *p* < 0.001), and (c) environmental context and resources (*OR* = 1.05; *p* = 0.017). Years of paediatric experience was also shown to be a significant predictor of higher knowledge scores, where those with more experience had greater odds of correct responses compared to those with 6–11 months experience (7–9 years: *OR* = 1.28, *p* = 0.013; 10 + years: *OR* = 1.34, *p* = 0.004). Nurses performed better in hospitals with greater ‘sepsis nurse funding’ (*OR* = 1.11; *p* = 0.002). The variation in knowledge scores between hospital sites was predominantly explained by the factors, clinical and ‘sepsis nurse funding’ variables. Figure 3 displays the odds ratios and 95% confidence intervals for all fixed effects variables (Fig. 3a) and demonstrates the reduction in variance attributed to hospital site (random intercept) when these fixed effects were adjusted for in the model (Fig. 3b and c).

**Table 3:** Multivariate Odds ratios (OR), 95% confidence intervals (95% CI) and *p*-values of the effects of the included variables on sepsis knowledge scores

Characteristic	OR^*1*^	95% CI^*1*^	***p***-value
Knowledge and beliefs	1.09	1.03, 1.15	0.001
Social influences	0.97	0.93, 1.02	0.293
Beliefs about capability and skills: Treatment	1.21	1.14, 1.27	< 0.001
Beliefs about capability and behaviour: Recognition, escalation, and management	1.04	0.98, 1.09	0.195
Environmental context and resources	1.05	1.01, 1.10	0.017
Age			
20–25 years	–	–	
26–30 years	1.02	0.87, 1.19	0.832
31–35 years	0.91	0.76, 1.09	0.302
36–40 years	0.99	0.81, 1.20	0.919
41–50 years	0.87	0.72, 1.04	0.135
51–60 years	0.92	0.73, 1.15	0.463
61+ years	1.11	0.74, 1.68	0.606
Length of time working in paediatrics			
6–11 months	–	–	
1–3 years	1.27	1.08, 1.49	0.004
4–6 years	1.18	0.99, 1.41	0.066
7–9 years	1.28	1.05, 1.56	0.013
10+ years	1.34	1.10, 1.63	0.004
Frequency of caring for a child with sepsis?			
Weekly	–	–	
Fortnightly	1.00	0.85, 1.17	0.986
Monthly	1.09	0.95, 1.26	0.234
Six monthly	1.12	0.94, 1.34	0.196
Yearly	1.17	0.84, 1.61	0.349
Don’t know	1.00	0.85, 1.18	0.984
Sepsis nurse funding	1.11	1.04, 1.18	0.002
Total number of clinicians	0.98	0.92, 1.05	0.514
Hospital SD	0.07		

^1^*OR* Odds Ratio, *CI* Confidence Interval, *Hospital SD* the standard deviation for the random intercept for hospital

1. Health Q. Career Structure 2019. Available from: https://www.health.qld.gov.au/employment/work-for-us/clinical/nursing-midwifery/career-structure

## Discussion

This study evaluated the implementation of a state-wide PSP by assessing the variables and factors contributing to ED nurse’s knowledge of paediatric sepsis recognition, escalation and management. The study captured data from a broad range of nurses and varying health care settings participating in a sepsis QI initiative. Respondents were from 14 metropolitan and regional EDs, of which four were dedicated paediatric departments and 10 were mixed adult-paediatric EDs in QLD, Australia.

This is the first study to explore and evaluate knowledge and factors contributing to implementation of a sepsis pathway, providing insight into the complex phenomena of knowledge translation [43]. The survey tool, designed from the TDF, was validated by an EFA and can serve future sepsis QI initiatives in other states and countries to assess KT and implementation, however further validation including confirmatory factor analysis on an larger cohort would enhance its validity. The study provided the following key findings: First, nurses predominately agreed or strongly agreed with statements contained in four of the five factors that reflect elements indicative of utilisation of the sepsis pathway. Second, variation in knowledge of paediatric sepsis existed across hospitals, despite participation in a state-wide QI project and the provision and supported implementation of a standardised sepsis pathway; variation in knowledge scores could be explained by the final two key findings, paediatric experience, ‘sepsis QI nurse funding’ and three identified factors. Third, the experience in paediatrics and dedicated ‘sepsis QI nurse funding’ focused on supporting the sepsis QI initiative, were key respondent and hospital site characteristics associated with improved implementation as measured by the knowledge score reflecting the key elements of the PSP. Finally, the factors (i) knowledge and beliefs, (ii) beliefs about capability and skills and (iii) environmental context and resources emerged as the strongest factors associated with the primary outcome, knowledge score.

### Translation of knowledge as a measure of sepsis pathway implementation

We used a knowledge assessment, composed of 15 questions targeting specific knowledge relevant for sepsis recognition, escalation, and management, formulated from the PSP. Time to initiation of sepsis treatment represents one of the strongest determinants of sepsis mortality in children [10, 44], yet studies have revealed major differences in compliance with sepsis bundles between sites using similar pathways, even when considering patient severity. Inter-institution variability in sepsis pathway knowledge and application can jeopardise sepsis recognition and treatment time [8, 9, 45], and contribute to suboptimal patient care [24, 46, 47]. Knowledge deficits may underpin the universal variability in sepsis pathway applicability resonating with previous literature articulating knowledge to be a key contributor to clinical pathway use [24]. Paediatric sepsis is particularly challenging because of the non-specific manifestation of sepsis and the relative rarity of sepsis compared to large numbers of febrile children being assessed in EDs, resulting in limited exposure for individual staff members [7]. In addition, a large proportion of children present to hospitals caring for adult and paediatric patients, staffed by a mixed, or primarily adult-experienced workforce. Hence, there is a need for evidence to guide improved strategies for the successful and sustainable implementation of sepsis pathways in children.

Our findings identified variability in sepsis knowledge, within and between sites [10, 11, 15], despite provision of a standardised, state-wide pathway and the related targeted education. These findings echo previous studies where average to low knowledge scores have been reported despite having a sepsis pathway and associated education in place [48–51]. Our study has identified that years of paediatric experience, ‘sepsis QI nurse funding’ and three identified factors associated with KT could explain a large proportion of variation in knowledge scores between sites, which can inform future sepsis initiatives.

### Factors associated with knowledge translation and implementation

Successful QI initiatives within emergency medicine require consideration of the variety of elements influencing clinician behaviour that may affect KT and subsequent implementation and practice change [52]. We explored a range of factors and identified three key factors which were significantly associated with improved knowledge of the PSP. Knowledge and beliefs, capability and skills, and environmental context and resources, emerged as key predictors of nurse’s knowledge scores. Furthermore, factors were independently indicative of KT to practice, by the reported high agreement scores, reflecting clinical practice insight, aligning with key elements of the PSP. KT is an underutilised phenomena in EDs [52], and focus on these three factors can inform future sepsis QI initiatives, campaigns and sepsis education to impact care globally, where previous efforts have reported limited success [14, 38, 53, 54].

Nurses in our study identified predominately positive agreement statements across four of the five factors indicating PSP utilisation, and 96% of nurses identified the pathway as a management resource, providing insight into implementation in clinical practice (Table 2; Supplementary Material 8). One factor, centred around the beliefs about capability and skills required to deliver treatment for paediatric sepsis, had comparatively lower ratings indicating that nurses need additional development to enhance confidence in skills required for managing paediatric sepsis. Similar findings have been identified in previous studies [51, 55], highlighting the importance of education initiatives focusing on kinaesthetic skill acquisition (and so, enhanced belief in capability) in training, especially where exposure to critically unwell children may otherwise be infrequent. In our study, only 35% of nurses reported caring for a child with sepsis as a weekly or fortnightly occurrence, highlighting the relatively low exposure to paediatric sepsis. The identified positive link between beliefs in capability and skills to sepsis knowledge should inform future QI and education initiatives to focus on increasing confidence in capability [51, 55–57] as a mechanism for enhancing knowledge translation and subsequent care for children.

### Institutional variables associated with knowledge translation and implementation

The variation we observed in knowledge scores between the 14 hospital sites was largely explained by dedicated ‘sepsis QI nurse funding’, irrespective of site, geographic and facility differences (Fig. 3c). A lack of provision of dedicated human resources has been identified as the greatest challenge in caring for critically ill paediatric patients, impacting nurse’s confidence in their capability [55]. Our identified link between confidence and knowledge informs future models, aiming to justify resources dedicated to paediatric sepsis QI initiatives. Our results emphasise the importance of specific training, education, tools and resources to increase nurse’s confidence about their capabilities around managing challenging presentations, [58, 59] such as paediatric sepsis. National and international sepsis guidelines have often achieved implementation success through use of sepsis leads and champions to advocate for education uptake and broad awareness [60, 61], with higher performing sites containing these components [62]. Whilst dedicated funding is idealistic, the complexities associated with ED nursing practice results in competing education requirements and funding priorities. Furthermore, challenges with healthcare budgets exist including, reduced funding sources and balancing cost efficiency for such initiatives. However if sepsis care is suboptimal, the costs and other burdens imposed on health systems are significant [49].

### Respondent characteristics associated with knowledge translation and implementation

We identified, years of paediatric experience as a significant predictor of increased knowledge. Previous study findings have also identified years of paediatric experience contributes to increased capability and confidence caring for paediatric patients [55]. While years of clinical experience cannot be replaced, we argue, based on our findings, there is a need for dedicated and ongoing resources targeting specialised paediatric sepsis education to enhance exposure; to supplement years of experience. These findings are supported in previous literature identifying that paediatric training and resources enhanced clinician’s exposure to and subsequent confidence in delivering care to critically unwell paediatrics [51, 63].

Importantly, surveys of students, and of the healthcare workforce have revealed relatively low levels of agreement and knowledge about sepsis, which contrasts with the fact that sepsis represents one of the leading diseases associated with preventable deaths across all age groups [49, 64]. In Australia, despite the fact that national sepsis standards are being developed, currently there are no standards or mandates for under-graduate, post-graduate, or facility specific training for ED nurses on sepsis [28, 55]. In Australia, a generalist model in undergraduate nursing curriculum [34] contrasts with that in the United Kingdom, which offers specialised paediatric nursing degrees. This may pose Australian nurses with a challenge as they are required to provide care across the lifespan, however children have significant differences in pathophysiology that requires specialist knowledge and training which is currently limited [55]. Indeed, a recent university study concluded knowledge of paediatric sepsis was as low as 8% in graduating nurses in Queensland, Australia [64]. The importance of specified paediatric training, including post-graduate qualifications, facility mandated courses and certification, requires exploration in the future development of health services and education [55, 63].

### Limitations

Knowledge of paediatric sepsis was used as the main outcome; however we did not assess whether increased knowledge was associated with improved treatment and outcomes in sepsis. In addition, we cannot conclusively attribute the results of the study to the implementation of the PSP due to the cross-sectional survey design. Furthermore, although this study identified important factors and variables associated with increased sepsis knowledge, we cannot assess whether there was an improvement in knowledge during the implementation of the PSP, as no baseline survey was conducted. A baseline survey would have provided valuable data and given greater certainty of KT, however given the complexities and challenges with ED nurse research including engagement with survey research, the multiple participating hospitals, competing priorities including the Coronavirus pandemic and funding, this was not possible. Thus, we explored the factors and variables contributing to knowledge, after the introduction of the PSP (and accompanying education), which may inform future QI initiatives. Participating hospitals may have chosen to support the sepsis QI initiative as part of regular business, resulting in voluntary contributions of time, which was not included in our calculations. We calculated ‘sepsis nurse funding’ as dedicated resourcing to lead the project and the predictive relationship of funding on knowledge score suggests that providing dedicated funding is of value. Two additional hospital sites were not included, as they had no sepsis-lead to run the survey. Independent data collection occurred locally, which may have contributed to response bias, unknown site confounding variables, and inconsistent instructions for completion alongside the biases that exist with self-reporting data [65]. We mitigated this risk by educating nurse leads at each site and providing an instruction script for consistent, supportive messaging. Each site was sent weekly response rates and reminders, the survey was available offline, and a strict inclusion criterion was created. A broad range of nurses were surveyed to include multiple perspectives and responses [39]. A sensitivity analysis of demographic details for partial responses that were excluded from analysis was undertaken to ensure no significant differences existed (Supplementary Material 6). Some sites had low response rates, with no ability to compare responders to non-responders, which impacts generalisability. Our sample was, however, heterogenous in nature with a large, combined site sample size representing many of the largest EDs within the state and is the largest paediatric sepsis implementation study conducted that assesses PSP implementation through KT.

## Conclusion and future direction

This is the first study to explore the implementation of a state-wide PSP, revealing important factors associated with knowledge of recognition, escalation and management of paediatric sepsis, reflecting key elements of the PSP. Findings have key implications for policy development and design of future QI initiatives, in particular, the significant contribution of dedicated sepsis funding to ensure consistency of education and associated pathway implementation. We have developed an internally validated survey tool and statistical model to be used in future health service models aiming to enhance healthcare delivery, that can be adapted, tested and applied to alternative cohorts. Future initiatives can use the key factors originally developed from the TDF, to design education packages and target performance improvement interventions that ensure sustainable KT strategies. Our study identified key factors influencing knowledge, offering a novel perspective to inform future interventions and evaluation of evidence-based care, to ensure successful implementation and sustainability of a PSP where global variability continues to exist.

## Supplementary Information

**Additional file 1.**

## Data Availability

The datasets used and/or analysed during the current study are available from the corresponding author on reasonable request.
