# Supplementary material for: Knowledge translation following the implementation of a state-wide Paediatric Sepsis Pathway in the emergency department- a multi-centre survey study
Source: BMC Health Serv Res. 2021 Oct 26;21:1161. doi: 10.1186/s12913-021-07128-2 (PMC8547904; doi:10.1186/s12913-021-07128-2)
Supplement: Supplementary file 1 — Additional file 1. [file 12913_2021_7128_MOESM1_ESM.docx]

***Supplementary Materials for:***

**Knowledge Translation following the implementation of a State-wide Paediatric Sepsis Pathway in the Emergency Department - a multi-centre survey study.**

Amanda Harley^1,2,3^, Luregn J. Schlapbach^2,4^, Paula Lister^5,6^ Debbie Massey^7^, Patricia Gilholm^2^, Amy, N. B. Johnston^1,8^.

1. School of Nursing, Midwifery and Social Work, The University of Queensland, QLD, Australia

2. Child Health Research Centre, The University of Queensland, and Paediatric Intensive Care Unit, Queensland Children’s Hospital, Brisbane, QLD, Australia

3. Department of Emergency Medicine, Gold Coast University Hospital, QLD, Australia

4. Pediatric and Neonatal Intensive Care Unit, Children`s Research Center, University Children`s Hospital Zurich, Zurich, Switzerland

5. Paediatric Intensive Care Unit, Sunshine Coast University Hospital, QLD, Australia

6. School of Medicine, Griffith University, QLD, Australia

7. School of Nursing and Midwifery, Southern Cross University, QLD Australia

8. Department of Emergency Medicine, Princess Alexandra Hospital, QLD Australia

**Correspondence**

Amanda Harley, State-wide Coordinator: Paediatric Sepsis Clinical Nurse Consultant

Queensland Children’s Hospital, Brisbane 4101, QLD Australia.

Email: Amanda.Harley@health.qld.gov.au

Phone: +61 7 3069 7484

**List of Supplementary Materials:**

1. **Paediatric Sepsis Pathway implementation dates, survey distribution dates, funded Paediatric Sepsis Pathway support and response rate (per site).**
2. **State-wide Paediatric Sepsis Pathway**
3. **Survey material distributed to respondents. Paediatric Sepsis Pathway: Knowledge Translation Survey for clinician.**
4. **Exploratory factor analysis method**
5. **Survey scoring**
6. **Sensitivity analysis of missing values**
7. **Proportion of correct responses for knowledge: Recognition, escalation and management of paediatric sepsis**
8. **Nurse responses to paediatric sepsis pathway utilisation**
9. **Box plot nurse Factor responses per site**

**Supplementary Material 1. Paediatric Sepsis Pathway implementation dates, survey distribution dates, funded Paediatric Sepsis Pathway support and response rate (per site).**

| **Site** | **PSP Commencement date** | **Survey distribution*** | **Sepsis nurse funding**** | **Response rate (%)***** |
| --- | --- | --- | --- | --- |
| 1 | 04-Feb-2019 | 3/12/19-14/1/20 | 1.15 | 39.0 |
| 2 | 16-Feb-2019 | 20/11/19- 1/1/20 | 6.01 | 45.7 |
| 3 | 11-Feb-2019 | 5/12/19-16/1/20 | 5.63 | 44.1 |
| 4 | 7-Aug-2018 | 13/9/19-25/10/19 | 11.27 | 46.4 |
| 5 | 23-Jan-2019 | 8/1/20-19/2/20 | 2.17 | 31.0 |
| 6 | 1-Mar-2019 | 20/11/19-1/1/20 | 0 | 45.6 |
| 7 | 04-Jul-2019 | 10/2/20-23/3/20 | 2.21 | 32.8 |
| 8 | 03-Apr-2019 | 4/10/19-15/11/19 | 9.17 | 40.7 |
| 9 | 14-Jan-2019 | 16/12/19-27/1/20 | 23.26 | 8.9 |
| 10 | 07-May-2019 | 26/11/19-7/1/20 | 0.88 | 36.6 |
| 11 | 04-Aug-2018 | 13/9/19-25/10/19 | 3.46 | 35.3 |
| 12 | 2-Dec-2018 | 26/9/19-7/11/19 | 5.04 | 80.0 |
| 13 | 10-Dec-2018 | 30/12/19-10/2/20 | 14.40 | 19.1 |
| 14 | 09-Mar-2019 | 6/12/19-17/1/20 | 5.25 | 45.8 |

*Survey responses received after this date were still included (<4%).

** Nurse lead funding calculated as ‘person months: Full time equivalent multiplied by months’ and weighted according to Queensland Health nursing salary (1)

*** Response rate calculated for participants who answered >80% of survey

**Supplementary Material 2: State-wide Paediatric Sepsis Pathway**

**
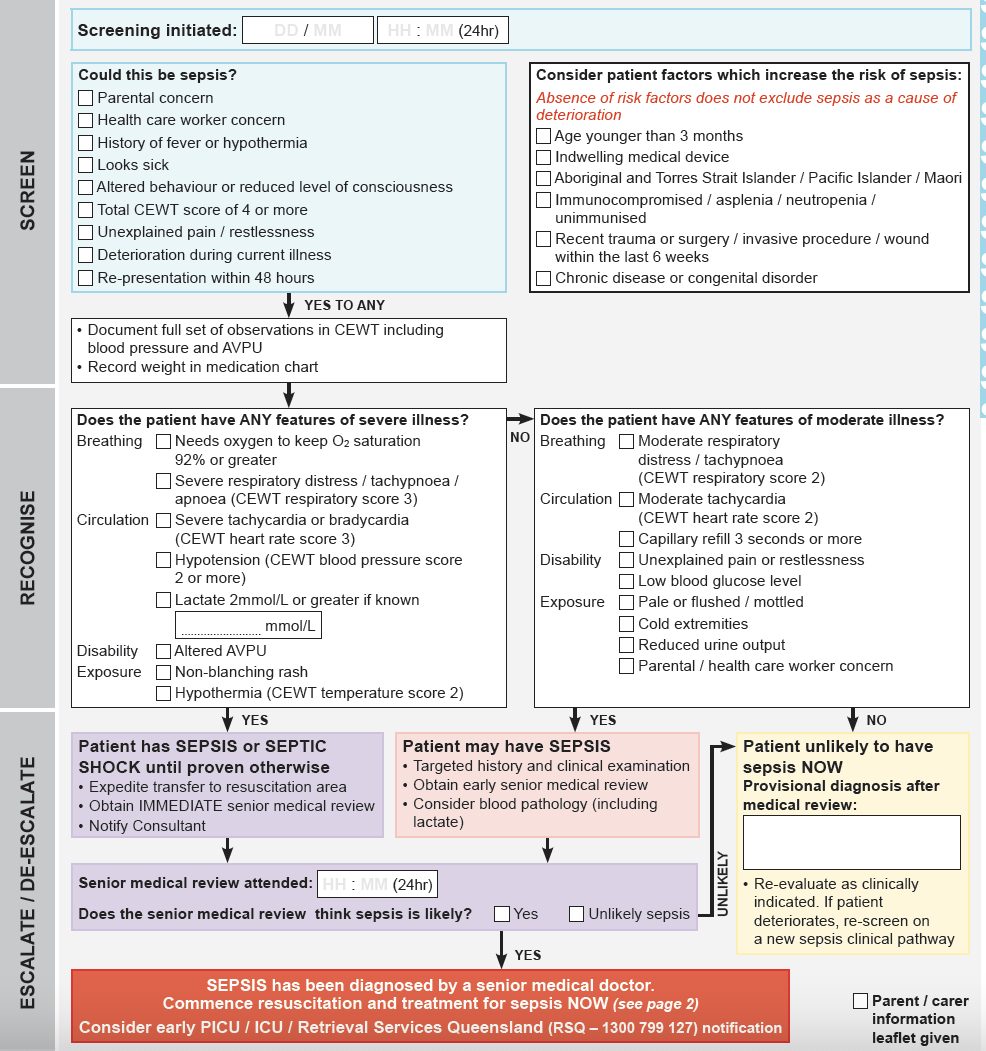
**

**
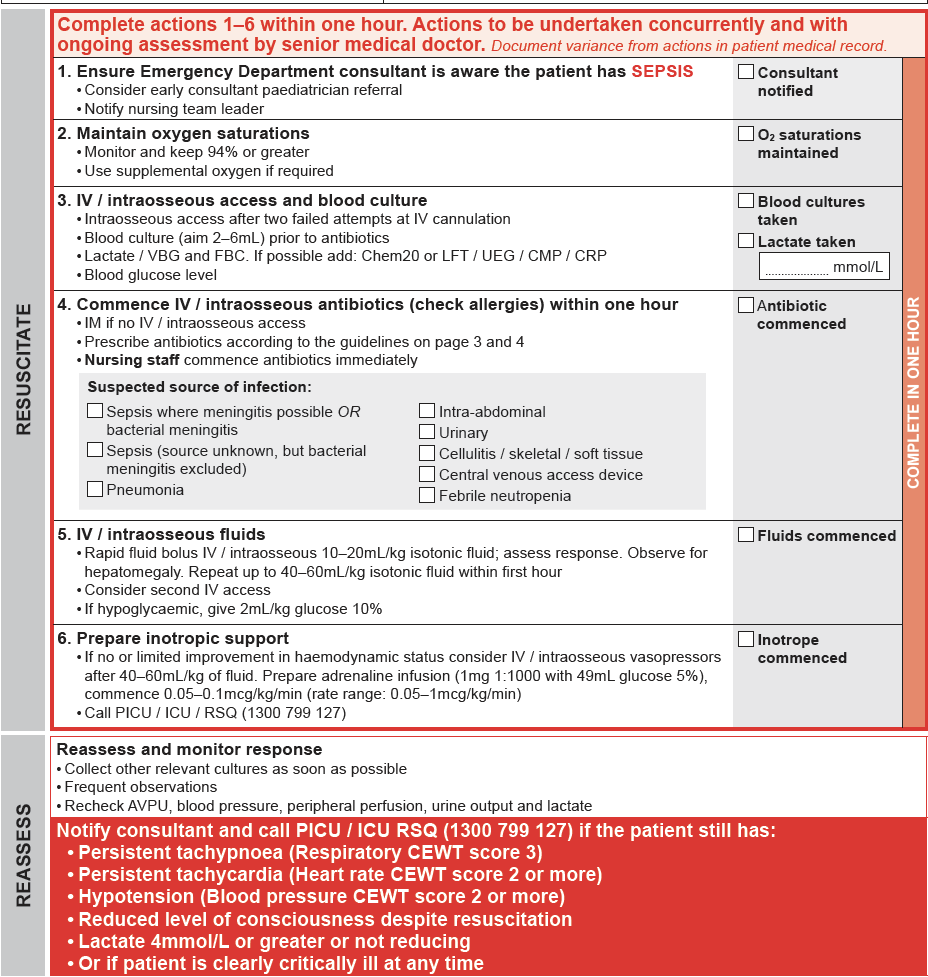
**

**Supplementary Material 3: Survey material distributed to respondents**

**Paediatric Sepsis Pathway: Knowledge Translation Survey for clinicians**

**Introduction:**

Paediatric sepsis is one of the leading causes of morbidity and mortality worldwide. Early recognition and management is key to improving outcomes. Your department has adopted the Paediatric Sepsis Pathway designed to supply clinicians with a tool given the pivotal role they play. The continued adverse outcomes experienced by sepsis highlights the importance to understand the knowledge and factors contributing to recognition, escalation and management of paediatric sepsis to develop new knowledge informing clinical practice.

**Aim**

Explore and describe the implementation a Paediatric Sepsis Pathway had on clinician’s knowledge, skills and confidence in recognising, escalating and managing paediatric sepsis in the Emergency Department (ED)?

**Secondary Aim**

Explore organisational factors that affect differences between clinicians’ knowledge, skills and confidence influencing the implementation of a Paediatric Sepsis Pathway and how these translate into maintenance.

**Your role:**

This survey will ask you questions pertaining to your knowledge, skills and confidence around recognising, escalating and managing paediatric patients with sepsis in your department. Information obtained in this survey is confidential, should take no longer than 15 minutes to complete and you will not be identified. Information collected will be analysed and used for evaluation. Your participation is voluntary and your decision to participate will not affect your current or future relationship with the organisation. If you do agree to participate you may withdraw from the study at any stage prior to submission.

**HREC/18/QRCH/167**

Any questions, queries or concerns please contact the Primary Investigator:

Amanda Harley: [amanda.harley@health.qld.gov.au](mailto:amanda.harley@health.qld.gov.au)

**Inclusion criteria:** ED nurses and doctors who have worked within their current ED caring for paediatrics for a minimum period of six months.

Please confirm that you have read the above information and are happy to complete the survey:

| - Yes | - No |  |
| --- | --- | --- |

Date and time of Survey Commencement: ______________________________

(DD-MM-YYYY HH:MM)

(Optional) Please confirm your Email Address:

__________________________________

This email address will be used to send you a repeat survey in six months.

**Demographics**

| **1.** **Which Hospital do you predominantly work at?** | - 1 - 2 - 3 - 4 - 5 - 6 | | - 7 - 8 - 9 - 10 - 11 - 12 | - 13 - 14 |
| --- | --- | --- | --- | --- |
|  |  | |  |  |
| **2. What kind of clinician are you? (Select all that are applicable)** | - Medical Intern - Medical Registrar - Medical Consultant - Clinical Facilitator | | - Enrolled Nurse - Endorsed Enrolled Nurse - Registered Nurse - Clinical Nurse - Nurse Unit Manager | - Nurse Educator - Clinical Nurse Consultant - Nurse Practitioner - Research Nurse - Other |
|  |  | |  |  |
| **3. What is your age?** | - 20 - 25 - 36- 40 | | - 26 – 30 - 41 – 50 | - 31 – 35 - 51-60 - 61+ |
| **4. How long have you been working in nursing /medicine?** | - 6-11 months - 7-9 years | | - 1-3 years - 10+ years | - 4-6 years |
|  |  | |  |  |
| **5. How long have you been working in paediatrics?** | - 6-11 months - 7-9 years | | - 1-3 years - 10+ years | - 4-6 years |
|  |  | |  |  |
| **6. How long have you been working in ED?** | - 6-11 months - 7-9 years | | - 1-3 years - 10+ years | - 4-6 years |
|  |  | |  |  |
| **7. How often do you care for a child who is diagnosed with sepsis?** | | - Weekly - Fortnightly - Monthly | | - Six monthly - Yearly - Don’t know |

**Knowledge**

*In the following section we are interested in exploring your knowledge of sepsis and the way in which it is recognised, escalated and managed within your Emergency Department.*

*Note: If unsure selected for any response, score is zero. Correct answers are indicated in* **bold.**

***Recognising***

| **8. What is sepsis?**  **(Select all that are applicable)** | - A severe Flu - **The body’s response to infection which injures tissues and organs.** - An uncontrollable fever causing rigors   *Note: If either or both answers are selected, one mark awarded.* | - **Infection with Systemic Inflammatory Response Syndrome** - Unsure |
| --- | --- | --- |
| **9. What presenting complaints would prompt you to initiate exploration of potential paediatric sepsis?**  **(Select all that are applicable)** | - Productive cough - **Re-presentation to health service** - **Parental concern** - **Altered behaviour** - **Reported history of fever or hypothermia** | - **Looks ‘sick’** - **Unexplained pain /restlessness** - Vomiting - Blanching rash - Runny nose - Diarrhoea - Unsure |
| **10. Why do you think sepsis in children can be difficult to recognise?**  **(Select all that are applicable)** | - **Lack of standardised tool for recognition** - It isn’t, you can instantly identify when hypotension is recorded - **Children’s Early Warning Tool not sensitive to sepsis** | - **Signs can be subtle** - **Key features can sometimes be explained by other causes** - Unsure |
| **11. Which of the following statements about lactate in children is true?**  **(Select all that are applicable)** | - Only a lactate above 4 is indicative of sepsis - Only arterial lactate samples are relevant | - **Any lactate above 2 in a child presenting with infection should be considered as a possible sign of sepsis** - **In a child where sepsis is treated a lactate should be obtained simultaneously with cultures** - Unsure |
| ***Escalating*** |  |  |
| **12. What is your understanding of ‘the golden hour’ in sepsis?**  **(Select all that are applicable)** | - Antibiotics must be given within an hour from triage time with any child with a suspected bacterial infection - All antibiotics must be given within an hour from triage time - **Targeted timeframe for all bundle elements to be commenced in sepsis** | - Refers only to cardiac and stroke patients - Timeframe to refer a patient to ICU - Unsure |
| **13. How do you escalate care for a child you think has sepsis? (Select all that are applicable)**  **(If you are a Medical Officer move to Q. 15)**  *Question 14 does not contribute to knowledge score* | - If the CEWT score is less than 4, wait and re-assess - I document my concerns in the triage notes for the next clinician to review - **I ask the supervising Senior Medical Officer (SMO) to review immediately**   *Note: If either or both answers are selected, one mark awarded.* | - Notify any available Medical Officer (MO) to review the patient within the next hour - **Seek input from a more senior or specialised clinician** - Initiate a review once a ‘E’ is scored on the CEWT or once obvious deterioration has occurred - Unsure |
| **14. How quickly does the child you are concerned about for sepsis get reviewed by a MO, based on existing infrastructure, policy and process?**  **(Select all that are applicable)**  Please specify the existing infrastructure, policy and process in place for MO review for sepsis at your hospital: | - Dictated by triage category - No tool currently in place for escalation time frame specific to sepsis - Depends entirely on current ED acuity and volume   _____________________________________________________ | - I request a review with/without documented concerns - Guided by CEWT score and correlating actions - None of the above (please specify below) - Unsure |
| ***Managing***  **15. What are the bundle components used in your hospital for paediatric patients diagnosed with sepsis?**  **(Select all that are applicable)**  Please specify which additional component(s) are used in your hospital: | - **Antibiotic administration** - **Fluid Bolus administration** - **Blood culture collection** - Unsure   _____________________________________________________ | - **Inotrope infusion if appropriate** - **Obtain lactate** - **Notify SMO** - Other (please specify below) |
|  |  |  |
|  |  |  |
| **16. Are you aware of a guideline/pathway to manage paediatric sepsis at your hospital?**  Please state which guideline(s) are used at your hospital: | - **Yes**   *Note: Paediatric Sepsis Pathway has to be listed to score 1.* | - No |
| **17. What typically guides your practice for managing children with sepsis?**  **(Select all that are applicable)**  Please specify what guides your practice for managing children with sepsis: | - Independent/individual clinician advice - Memory of Evidence Based Practice - Depends on presentation - Clinical experience - Other   *Note: Paediatric Sepsis Pathway has to be selected or written here to score 1. If additional answers are selected, no extra marks awarded or subtracted.* | - ED HHS specific guideline   - **QLD Paediatric Sepsis Pathway**   - Combined Adult and Paediatric guideline |
| **18. Does your ED deliver Inotropes in paediatric sepsis?** | - **Yes (Go to Q.19)** | - No (Go to Q.20) |
| **19. What is your understanding of the care requirements for a child receiving inotropes?**  **(Select all that are applicable)** | - **Requires retrieval or admission to ICU** - **Requires 1-1 nursing care** - **Can be given via peripheral or intraosseous line** - Cannot be started in ED | - Must be given via central access - **Requires blood pressure/MAP monitoring for titration** - Unsure |
|  |  |  |
|  |  |  |
|  |  |  |
|  |  |  |
| **(If you are a Medical Officer move to Q. 21)** |  |  |
| **20. What resources do you currently use to help you administer antibiotics to paediatric patients with sepsis in the ED?**  **(Select all that are applicable)** | Australian Injectables Handbook  Local guideline  (please specify name)  *Note: If Paediatric Sepsis Pathway written, scored as correct*  Other hospital’s guideline/procedure  (please specify name)  *Note: If Paediatric Sepsis Pathway written, scored as correct*  **Paediatric Sepsis Pathway** | Seek senior/speciality colleague support  Limited resources required- clinical experience guides me  *Note: If above selected, 0.5 is the maximum score awarded and only if the correct response was also selected*  Online resources (please specify) ______________________ |
| **21. What is the recommended amount of fluid that is given for initial resuscitation of septic shock in children?**  **(If you are a Medical Officer move to Q. 24)**  **22. Which resources would you utilise to guide preparation of an Adrenaline infusion for paediatrics? (Select all that are applicable)** | - 20ml/kg in 5ml/kg aliquots - **Up to 40-60ml/kg in 10-20 ml/kg aliquots** - 20ml/kg is maximum amount before inotropes are commenced - Phone PICU for advice - Seek input/assistance from ED pharmacist - **Consult Paediatric Sepsis Pathway** - Escalate to PICU for transfer for commencement in PICU | - Continue to give fluids until MAP normalised - Inotropes should be commenced anytime in patients that look peri-arrest - Unsure - Support from Australian Injectables handbook   Local guideline  (please specify name)  *Note: If Paediatric Sepsis Pathway written, scored as correct*  Other hospital’s guideline/procedure  (please specify name)  *Note: If Paediatric Sepsis Pathway written, scored as correct*   - Unsure |
|  |  |  |
| **23. What strength Adrenaline and equipment is required for an Adrenaline infusion for paediatrics? (Select all that are applicable)** | - Adrenaline 1:10000, 3-way tap, 50 ml syringe and 5% dextrose | - **Adrenaline 1:1000, 3-way tap, 50 ml syringe and 5% dextrose** - Adrenaline 1:100000, 3-way tap, 50 ml syringe and 5% dextrose - Unsure |

**Circle the answer from 1 ‘strongly disagree’ to 7 ‘strongly agree’**

*In the next section we are interested in exploring your confidence and skills in recognising, escalating and managing paediatric patients with sepsis in the Emergency Department.*

| **1** | **2** | **3** | **4** | **5** | **6** | | | | **7** | | | |
| --- | --- | --- | --- | --- | --- | --- | --- | --- | --- | --- | --- | --- |
| **Strongly Disagree** | **Disagree** | **Slightly Disagree** | **Neutral** | **Slightly Agree** | **Agree** | | | | **Strongly Agree** | | | |
| **Items** | | | | | **Ranking** | | | | | | | |
| **Recognising:** | | | | |  |  |  |  | |  |  |  |
| 24. I am confident that I could recognise sepsis in a paediatric patient | | | | | 1 | 2 | 3 | 4 | | 5 | 6 | 7 |
| 25. **(If you are a Nurse move to Q. 26)**  I feel confident in making the decision that a patient does NOT have sepsis | | | | | 1 | 2 | 3 | 4 | | 5 | 6 | 7 |
| **Escalating:** | | | | |  |  |  |  | |  |  |  |
| 26. I am sufficiently trained to respond to sepsis in a paediatric patient | | | | | 1 | 2 | 3 | 4 | | 5 | 6 | 7 |
| 27. I have a clear plan of how I will escalate care of a patient with suspected sepsis following the Paediatric Sepsis Pathway | | | | | 1 | 2 | 3 | 4 | | 5 | 6 | 7 |
| **Managing:** | | | | |  |  |  |  | |  |  |  |
| 28. **(If you are a Medical Officer move to Q. 29)**  I am confident that I can set up an Adrenaline infusion in a time critical situation | | | | | 1 | 2 | 3 | 4 | | 5 | 6 | 7 |
| 29. I have the skills to calculate and deliver a fluid bolus to a paediatric patient as rapidly as their condition demands | | | | | 1 | 2 | 3 | 4 | | 5 | 6 | 7 |
| 30. When I have to prescribe or deliver an intravenous antibiotic I feel comfortable using the guide on the Paediatric Sepsis Pathway | | | | | 1 | 2 | 3 | 4 | | 5 | 6 | 7 |

**Circle the answer from 1 ‘very difficult to 7 ‘very easy’**

*In the next section we are interested in exploring your perception of your organisation when recognising, escalating and managing paediatric patients with sepsis in the Emergency Department.*

| **1** | **2** | **3** | **4** | **5** | **6** | | | | **7** | | | |
| --- | --- | --- | --- | --- | --- | --- | --- | --- | --- | --- | --- | --- |
| **Very Difficult** | **Difficult** | **Slightly Difficult** | **Neutral** | **Slightly Easy** | **Easy** | | | | **Very Easy** | | | |
| **Items** | | | | | **Ranking** | | | | | | | |
| **Recognising & Escalating:** | | | | |  |  |  |  | |  |  |  |
| 31. **(If you are a Medical Officer move to Q. 32)**  Escalating my concerns to a Senior Medical Officer that a child could have sepsis is: | | | | | 1 | 2 | 3 | 4 | | 5 | 6 | 7 |
| 32. **(If you are a Nurse move to Q. 33)**  Escalating my concerns to ICU that a child has sepsis is: | | | | | 1 | 2 | 3 | 4 | | 5 | 6 | 7 |
| **Managing:** | | | | |  |  |  |  | |  |  |  |
| 33. For me, seeking support from colleagues when uncertain about the management of paediatric sepsis is: | | | | | 1 | 2 | 3 | 4 | | 5 | 6 | 7 |

**Circle the answer from 1 ‘strongly disagree’ to 7 ‘strongly agree’**

*In the next section we are interested in exploring your experience with organisational structures and the way in which care is given encompassing recognising, escalating and managing paediatric patients with sepsis in the Emergency Department.*

| 1 | **2** | **3** | **4** | **5** | **6** | | | | **7** | | | |
| --- | --- | --- | --- | --- | --- | --- | --- | --- | --- | --- | --- | --- |
| **Strongly Disagree** | **Disagree** | **Slightly Disagree** | **Neutral** | **Slightly Agree** | **Agree** | | | | **Strongly Agree** | | | |
| **Items** | | | | | **Ranking** | | | | | | | |
| **Recognising:** | | | | |  |  |  |  | |  |  |  |
| 34. Recognising sepsis can be challenging and the clinical signs are not always obvious | | | | | 1 | 2 | 3 | 4 | | 5 | 6 | 7 |
| 35. My hospital provides doctors and nurses with sufficient training to recognise and manage treatment for paediatric patients with sepsis | | | | | 1 | 2 | 3 | 4 | | 5 | 6 | 7 |
| **Escalating:** | | | | |  |  |  |  | |  |  |  |
| 36. Senior staff in the organisation in which I work are willing to listen to my problems with escalating care and finding placement for paediatric patients with sepsis | | | | | 1 | 2 | 3 | 4 | | 5 | 6 | 7 |
| 37. I can count on gaining support from doctors and nurses to respond when I suspect a patient has sepsis | | | | | 1 | 2 | 3 | 4 | | 5 | 6 | 7 |
| **Managing:** | | | | |  |  |  |  | |  |  |  |
| 38. I can remember the important steps of care for paediatric patients with sepsis | | | | | 1 | 2 | 3 | 4 | | 5 | 6 | 7 |
| 39. In my ED, I think there are all the necessary resources available to efficiently manage paediatric sepsis | | | | | 1 | 2 | 3 | 4 | | 5 | 6 | 7 |
| 40. In my hospital, there is a good collaboration between Paediatrics or retrieval teams and the ED when accepting patients with sepsis who require ongoing care | | | | | 1 | 2 | 3 | 4 | | 5 | 6 | 7 |
| 41. Delivering sepsis care following the pathway is a high priority of mine when working in the ED | | | | | 1 | 2 | 3 | 4 | | 5 | 6 | 7 |

**Circle the answer from 1 ‘strongly disagree’ to 7 ‘strongly agree’**

*In the next section we are interested in exploring your knowledge and beliefs surrounding recognising, escalating and managing paediatric patients with sepsis in the Emergency Department*.

| 1 | **2** | **3** | **4** | **5** | **6** | | | | **7** | | | |
| --- | --- | --- | --- | --- | --- | --- | --- | --- | --- | --- | --- | --- |
| **Strongly Disagree** | **Disagree** | **Slightly Disagree** | **Neutral** | **Slightly Agree** | **Agree** | | | | **Strongly Agree** | | | |
| **Items** | | | | | **Ranking** | | | | | | | |
| **Recognising:** | | | | |  |  |  |  | |  |  |  |
| 42. It is my responsibility to recognise sepsis in the ED | | | | | 1 | 2 | 3 | 4 | | 5 | 6 | 7 |
| 43. I am often triggered to think about sepsis in children by the level of parental concern voiced | | | | | 1 | 2 | 3 | 4 | | 5 | 6 | 7 |
| 44. My ED recommends the inclusion and involvement of parents in assessment and clinical management of paediatric patients with sepsis | | | | | 1 | 2 | 3 | 4 | | 5 | 6 | 7 |
| **Escalating:** | | | | |  |  |  |  | |  |  |  |
| 45. **(If you are a Medical Officer move to Q. 47)**  Obtaining an immediate senior medical review for a child suspected of sepsis is something I feel confident to do | | | | | 1 | 2 | 3 | 4 | | 5 | 6 | 7 |
| 46. For me, reporting about patients that MAY have sepsis to the Medical Officer is beneficial in expediating care | | | | | 1 | 2 | 3 | 4 | | 5 | 6 | 7 |
| 47. If I deliver sepsis care following the Paediatric Sepsis Pathway I will feel satisfied in the care I am delivering to my patient | | | | | 1 | 2 | 3 | 4 | | 5 | 6 | 7 |
| **Managing:** | | | | |  |  |  |  | |  |  |  |
| 48. Delivering the treatment bundle on the sepsis pathway is part of my role as a clinician | | | | | 1 | 2 | 3 | 4 | | 5 | 6 | 7 |
| 49. If I deliver care within one hour following the treatment bundle on the sepsis pathway this will result in a better patient outcome. | | | | | 1 | 2 | 3 | 4 | | 5 | 6 | 7 |
| 50. Delivering sepsis care following the Paediatric Sepsis Pathway is something I do as part of routine patient management | | | | | 1 | 2 | 3 | 4 | | 5 | 6 | 7 |

**Supplementary Material 4: Exploratory factor analysis method:**

An exploratory factor analysis using oblique rotation was performed on the 25 items that make up the participants’ perceptions section of the survey. A combination of statistical criteria and clinical judgement were used to determine the optimal number of factors (2). Inspection of the factor loadings, which represent the strength of the relationship between each item and each factor (1), was performed to determine if the factors met the following three criteria:

1. a minimum of 3 items contributing to the factor, measured by factor loadings greater than or equal to .3,
2. a minimum factor loading of .3 was used as a cut-off to determine if the item contributed to the factor,
3. questions that had cross loadings (loadings of .3 or greater on more than 1 factor) or had low loadings on all factors were removed to clarify the interpretation of the factors (2).

Parallel analysis (3) uncovered a four-factor solution. The four-factor solution was inspected and compared with a five-factor solution that was ultimately retained, as the additional factor that appeared was deemed to be clinically relevant.

Four survey questions were removed as they exhibited cross-loadings or had no strong factor loadings. These five factors explained a total of 58% of the variation in the responses and correlations between the factors ranged from 0.33 to 0.66.

Factor scores are variables describing how each individual would score on the factors if they were observed (3). The factor scores were extracted using Bartlett’s method (2). In this method, factor score coefficients (similar to regression coefficients) are created based on the factor loadings and the correlations between the items. The individual’s standardised score for each item is then multiplied by the corresponding factor score coefficient and summed to create an estimate for each factor (4). By creating the factor scores in this way, each item is weighted by its factor loading, whereby items with larger factor loadings will have more weight than items with lower loadings.

**Supplementary Material 5: Survey scoring**

Questions allowing multiple correct answers were partially scored, with the following scoring criteria; where all correct responses were selected and all incorrect responses were not selected, the participant received 1 point for the question. Where 80% or more of the correct responses were selected and only 20% or fewer of the incorrect responses were selected then the participant received a partial mark of 0.5, otherwise they received a zero for the question. If responses had more than one response when not indicated, the variable was coded as incorrect. If the sum of correct responses resulted in a partial mark (e.g. 19.5), the score was rounded up to a whole number, to comply with the assumptions of binomial logistic regression modelling (4).

If responses had more than one response selected within the Likert scale, the ‘lower agreement response’ was selected within the Likert scale

**Supplementary Material 6: Sensitivity analysis of missing values**

An investigation between participants who had completed 80% or more of the total survey and those that had completed less than 80% was undertaken and revealed no significant differences between these groups on any of the demographic variables, except age group, where there were significantly fewer over-50 year old’s who completed 80% or more of the survey. To avoid imputation of a large number of missing responses, only the sample that completed more than 80% of the survey were included in the analysis (N =544). An additional 10 nurses were excluded from the final analysis as they did not respond to key demographic questions (N= 534).

**Difference between responders who had completed 80% or more of the total survey and those that had completed less than 80% of the total survey**

| **Characteristic** | **Completed less than 80%,**  N = 132*^1^* | **Completed 80% or more**,  N = 544*^1^* | **p-value***^2^* |
| --- | --- | --- | --- |
| Hospital type |  |  | 0.077 |
| Quaternary | 5 (3.8%) | 52 (9.6%) |  |
| Specialised | 33 (25%) | 144 (26%) |  |
| Mixed | 94 (71%) | 348 (64%) |  |
| Age group |  |  | 0.010 |
| 20-35 years | 72 (55%) | 321 (59%) |  |
| 36-50 years | 32 (24%) | 161 (30%) |  |
| 51+ years | 28 (21%) | 62 (11%) |  |
| Nursing experience |  |  | 0.693 |
| 6 months – 3 years | 25 (19%) | 104 (19%) |  |
| 4-6 years | 38 (29%) | 138 (26%) |  |
| 7-9 years | 16 (12%) | 86 (16%) |  |
| 10+ years | 53 (40%) | 213 (39%) |  |
| Unknown | 0 | 3 |  |
| Paediatric experience |  |  | 0.111 |
| 6-11 months | 22 (17%) | 69 (13%) |  |
| 1-3 years | 38 (29%) | 149 (28%) |  |
| 4-6 years | 21 (16%) | 125 (23%) |  |
| 7-9 years | 13 (9.8%) | 76 (14%) |  |
| 10+ years | 38 (29%) | 117 (22%) |  |
| Unknown | 0 | 8 |  |
| ED experience |  |  | 0.224 |
| 6-11 months | 13 (9.9%) | 45 (8.4%) |  |
| 1-3 years | 38 (29%) | 149 (28%) |  |
| 4-6 years | 24 (18%) | 132 (25%) |  |
| 7-9 years | 15 (11%) | 84 (16%) |  |
| 10+ years | 41 (31%) | 128 (24%) |  |
| Unknown | 1 | 6 |  |
| Frequency of care for child with sepsis |  |  | 0.404 |
| Less often | 60 (45%) | 223 (41%) |  |
| Often | 72 (55%) | 321 (59%) |  |
| *^1^*Statistics presented: n (%)  *^2^*Statistical tests performed: chi-square test of independence | | | |

Note: Some original categories have been combined due to low cell counts

**Supplementary Material 7: Proportion of correct responses for knowledge: Recognition, escalation and management of paediatric sepsis**


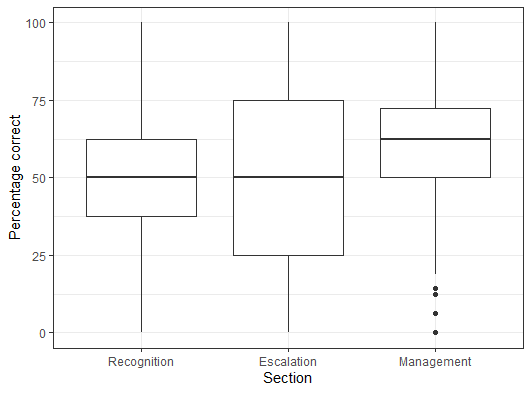


*Supplementary material 7: The centre line for each box plot is the median value, the upper-lower limits of the box are the 1^st^ and 3^rd^ quartile. The whiskers correspond to the maximum and minimum points that are 1.5* the IQR from the 1^st^ and 3^rd^ quartiles. Data that exceed these limits are considered outliers and plotted individually.*

**Supplementary Material 8: Nurse responses to paediatric sepsis pathway utilisation**

Q.14 How quickly does the child you are concerned about for sepsis get reviewed by a MO, based on existing infrastructure, policy and process?


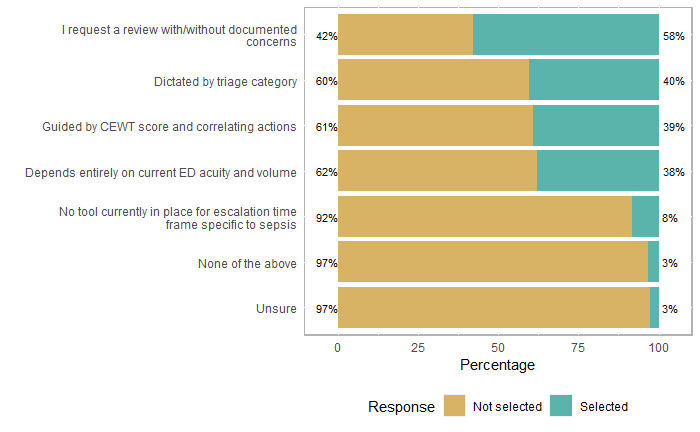


17. What typically guides your practice for managing children with sepsis? (Select all that are applicable)


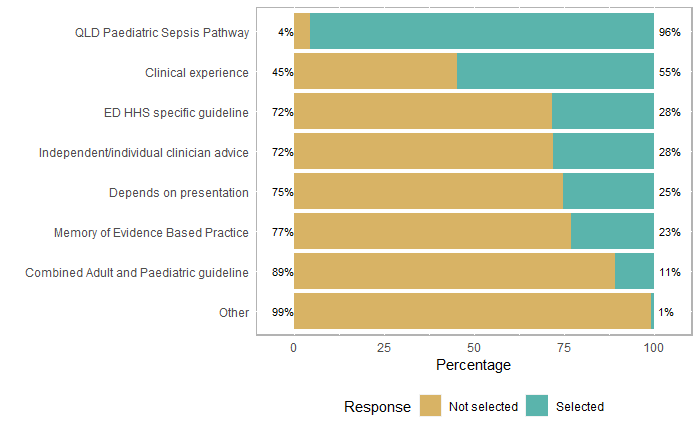


20. What resources do you currently use to help you administer antibiotics to paediatric patients with sepsis in the ED? (Select all that are applicable)


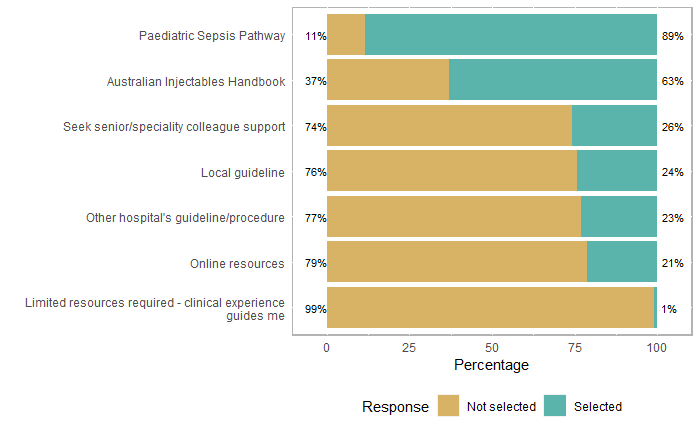


22. Which resources would you utilise to guide preparation of an Adrenaline infusion for paediatrics? (Select all that are applicable)


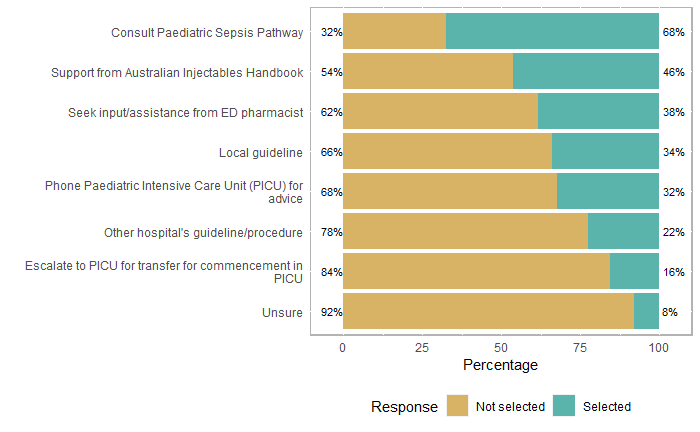


**Supplementary Material 9: Box plot Factor nurse responses per site:**

The following five figures outline nurse’s responses to each independent factor, per hospital site.

**Factor 1: “Knowledge and beliefs about paediatric sepsis and pathway application”**


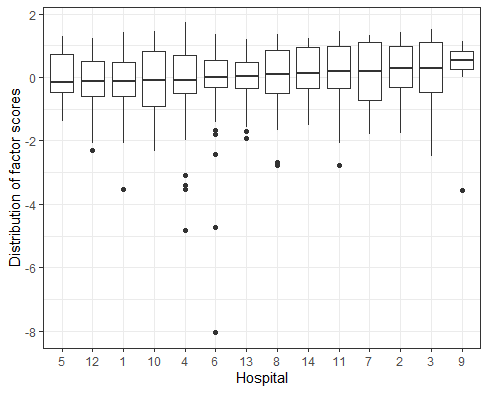


**Factor 2 “****Social influences when recognising, escalating and managing paediatric sepsis”**


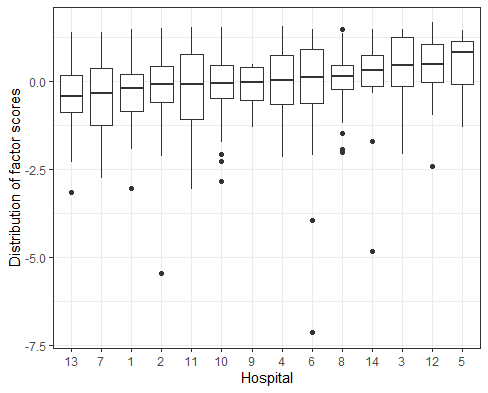


**Factor 3: “Beliefs about capability and skills delivering treatment for paediatric sepsis”**


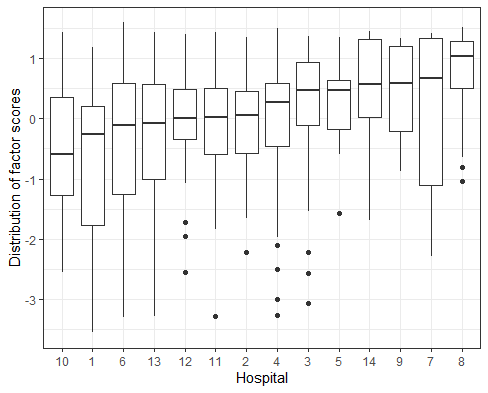


**Factor 4 “Beliefs about capability and behaviour in recognising, escalating and managing paediatric sepsis”**


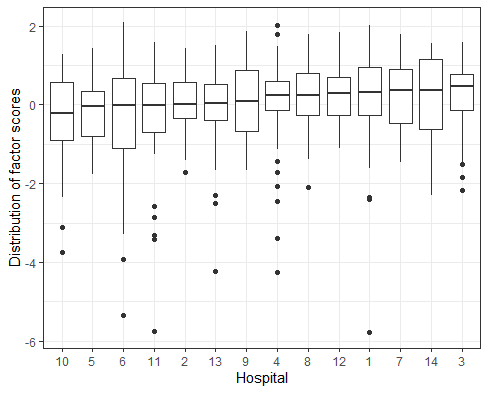


**Factor 5 “ Environmental context and resources in the ED for recognising, escalating and managing paediatric sepsis”**


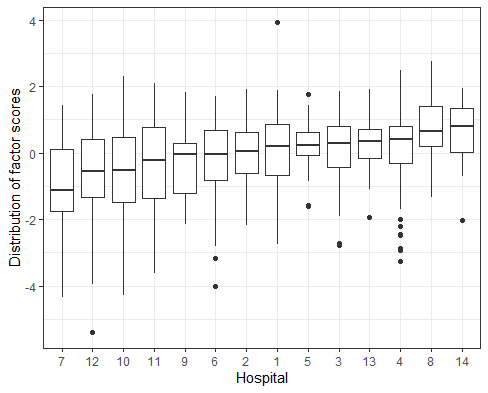


**References**

1. Health Q. Nursing Stream Wage 2020 [Available from: <https://www.health.qld.gov.au/hrpolicies/wage-rates/nursing>.

2. Yong AG, Pearce S. A Beginner’s Guide to Factor Analysis: Focusing on Exploratory Factor Analysis. Tutorials in quantitative methods for psychology. 2013;9(2):79-94.

3. Flora DB, LaBrish C, Chalmers RP. Old and New Ideas for Data Screening and Assumption Testing for Exploratory and Confirmatory Factor Analysis. Front Psychol. 2012;3:55-.

4. Tabachnick BG, Fidell LS, Ullman JB. Using multivariate statistics. Boston: Pearson/Allyn & Bacon; 2007.
